# Editorial: Novel and promising laboratory biomarkers for allergic disease diagnosis and prognosis: clinical applicability

**DOI:** 10.3389/falgy.2025.1771107

**Published:** 2026-01-14

**Authors:** Henry Velazquez-Soto, Xin Wang, María C. Jiménez-Martínez

**Affiliations:** 1 Department of Immunology, Research Unit, Institute of Ophthalmology “Conde de Valenciana Foundation”, Mexico City, Mexico; 2 Jeff and Penny Vinik Center for Translational Immunology Research, Division of Allergy and Clinical Immunology, Brigham and Women’s Hospital, Boston, United States; 3 Department of Medicine, Harvard Medical School, Boston, United States; 4 Department of Biochemistry, Faculty of Medicine, National Autonomous University of Mexico, Mexico City, Mexico

**Keywords:** allergy biomarkers, allergy diagnosis, allergy genetics, drug allergy, rhinitis diagnosis, serum sickness

Allergic diseases represent a growing and heterogeneous group of immune-mediated disorders that continue to challenge conventional diagnostic and prognostic frameworks. While IgE-based assays, skin testing, and clinical history remain central to allergy practice, these tools often provide limited insights into disease heterogeneity, severity, prognosis, and therapeutic response. Increasingly, there is a need for laboratory biomarkers and diagnostic methodologies that move beyond classification toward prediction and personalization. This Research Topic was launched to highlight innovative advances addressing this unmet clinical need.

This collection reflects both the rapid evolution of biomarker research in allergy and the strong engagement of the clinical and scientific community. The contributions span point-of-care diagnostics, molecular and genetic biomarkers, proteomics, computational analyses, and mechanistic immunology.

Clinical translation is a unifying theme throughout the Topic. The comprehensive review “Unravelling allergic rhinitis: exploring pathophysiology, advances in treatment, and future directions” by Singh et al. provides a clinically oriented synthesis of disease mechanisms and emerging therapeutic strategies, highlighting where biomarker-driven approaches may enable more individualized management. Building on this, the study by Dai et al. “Identification of diagnostic signature, molecular subtypes, and potential drugs in allergic rhinitis based on an inflammatory response gene set” demonstrates how transcriptomic profiling can refine disease stratification and identify candidate therapeutic targets, reinforcing the promise of molecular precision in common allergic conditions.

Several articles address practical diagnostic innovation with direct relevance to daily clinical workflows. “Allergy diagnostic performance of Factcheck 20 Atopy” by Nösslinger et al. evaluates a rapid point-of-care assay that may complement traditional laboratory testing, particularly in settings where access to specialized diagnostics is limited or results are equivocal. In parallel, “Efficacy of the PEN-FAST score in a French cohort of patients with reported allergy to penicillins” by Hanniet et al. highlights how structured clinical tools, when appropriately validated, can safely streamline allergy assessment, reduce unnecessary testing, and support antibiotic stewardship.

The growing importance of genetic and molecular diagnostics in allergy is illustrated by the work of Wetherby et al. “Identification of an elusive SERPING1 deletion in a family with hereditary angioedema type I utilizing soft clipping”. This study highlights the utility of advanced sequencing and analytical strategies in resolving diagnostically challenging cases, thereby enabling accurate diagnosis and effective long-term management in rare but clinically significant allergic disorders.

Innovation in computational and systems-level approaches is represented by Zhong et al. in the article “Machine learning-based screening of asthma biomarkers and related immune infiltration”, which applies data-driven methods to identify immune signatures associated with asthma. Such approaches exemplify a shift away from single-parameter biomarkers toward multidimensional models with potential utility in disease phenotyping, prognosis, and therapeutic selection.

Mechanistic understanding remains essential for meaningful translation. “TRIM28 mediates Mettl5 ubiquitination to promote Th2 polarization” by Miao et al. provides insight into intracellular regulatory pathways governing Th2 bias, a hallmark of many allergic diseases. By linking molecular mechanisms to immune polarization, this work lays a biological foundation for future biomarker and therapeutic development.

The expanding role of high-throughput proteomics in clinical allergy is highlighted by Zhu et al. in the “Case Report: Serum sickness induced by dupilumab—clinical insights and Olink proteomic analysis”. Although based on a single case, this contribution illustrates how multiplex protein profiling can capture systemic immune changes associated with biologic therapies, offering a glimpse into future strategies for treatment monitoring and adverse event characterization.

Finally, “Diagnostic tests for progestogen hypersensitivity” by Alonso Bello et al. addresses a clinically challenging and often underrecognized condition, emphasizing the need for standardized, evidence-based laboratory diagnostics and improved clinical awareness.

Taken together, this Research Topic reflects a field in transition from descriptive diagnostics toward predictive, mechanism-informed, and clinically actionable biomarker strategies. While further validation, standardization, and larger and more robust clinical studies are needed before many of these approaches can be fully integrated into routine practice, the work presented here outlines clear trajectories for translational progress.

We thank all authors for their innovative contributions, the reviewers for their rigorous evaluations, and the allergy community for engaging with this collection. We hope this Research Topic serves both as a snapshot of current advances and as a catalyst for continued efforts to improve diagnostic precision and patient outcomes in allergic diseases.

